# Sacral Agenesis: Late Presentation and the Psychological Impact of Delayed Diagnosis

**DOI:** 10.7759/cureus.47456

**Published:** 2023-10-22

**Authors:** Jyoti Bhutani, Vinayak Rengan, Vishnu Pansari, Deerush Kannan

**Affiliations:** 1 Paediatrics, SMS Medical College, Jaipur, IND; 2 Paediatric Surgery, SMS Medical College, Jaipur, IND; 3 Urology, Apollo Hospitals, Chennai, IND

**Keywords:** constipation, prenatal screening, delayed diagnosis, urinary dysfunction, sacral agenesis

## Abstract

Sacral agenesis (SA) is a rare condition characterized by the absence of one or more lower sacral vertebral bodies. In India, children with this condition often present late with symptoms primarily related to urinary and bowel dysfunction. Maternal diabetes is the only confirmed risk factor, significantly elevating the incidence rate. We discuss a case of a nine-year-old female who presented to the pediatric outpatient department (OPD) with chronic constipation and urinary retention, having experienced symptoms since infancy. Initial investigations at peripheral hospitals had yielded no clear diagnosis, leading to undue psychological distress to the child and family. The child had been born to a mother with diabetes mellitus during pregnancy. Physical examination revealed mild dehydration, anemia, and sacral dimpling. Further evaluation showed renal injury and SA confirmed by MRI, along with other associated findings.

This case report highlights the importance of early diagnosis and intervention in pediatric SA, especially given the risk of renal disease progression. The treatment in this case included clean intermittent self-catheterization (CIC), dietary management, and counseling on renal health. Crucially, uncovering the root cause provided immense psychological relief to the child and her family. Pediatric SA remains a diagnostic challenge, often leading to psychological distress in affected individuals who present late. Early recognition and comprehensive management are crucial, especially in cases associated with maternal diabetes, to mitigate the risk of renal complications and improve the overall quality of life for affected children.

## Introduction

Sacral agenesis (SA) is a rare condition, and it is characterized by the absence of part or all of two or more lower sacral vertebral bodies. It is part of the caudal regression syndrome (CRS) spectrum. Its extreme rarity (one in 25,000 live births) means that its diagnosis is often missed at initial presentations [1]. In India, clinical experience has shown that most patients with this condition present at a very late age (early or mid-childhood). Individuals who seek medical attention at a later stage often present to the outpatient clinic exhibiting symptoms of urinary dysfunction and bowel habit disturbances, predominantly constipation. Maternal diabetes is the only confirmed risk factor for this condition. In children with diabetic mothers, the incidence is one in 350, representing a nearly 200-fold increase compared to the usual rate [2].

## Case presentation

Case history and presentation

A nine-year-old girl presented to the pediatrics outpatient department (OPD) with complaints of chronic constipation and urinary retention, with symptoms that she had carried since infancy. On arrival, the patient's bladder was catheterized with a 10 French Foley catheter and more than 300 ml of urine was drained. She had a history of poor urinary control with overflow incontinence since infancy. The patient had been investigated at peripheral hospitals since birth but no obvious neurological deficit or clinical finding could be elicited. No uroflowmetry or imaging studies had been performed prior to admission at our unit. The lack of a proper diagnosis led to the patient being branded as having a psychosomatic illness. This had caused intense psychological distress to the patient and her mother. The child psychologist noted anxiety and depressive symptoms in the patient. 

The patient was the firstborn of a non-consanguineous marriage, delivered via normal vaginal birth. The mother gave a history of poorly controlled maternal diabetes mellitus. She had taken periconceptional folic acid. There were no records of antenatal scans. On examination, the patient had mild dehydration and anemia. The blood pressure and other vital examination findings were within normal limits. Her height and weight were appropriate for her age. Developmental milestones had been attained at appropriate ages. There was no obvious neurological deficit and the gait was normal. There was mild excoriation in the perineum likely due to constant urinary dribbling. There was also dimpling in the sacral region.

Investigations

**Figure 1:**
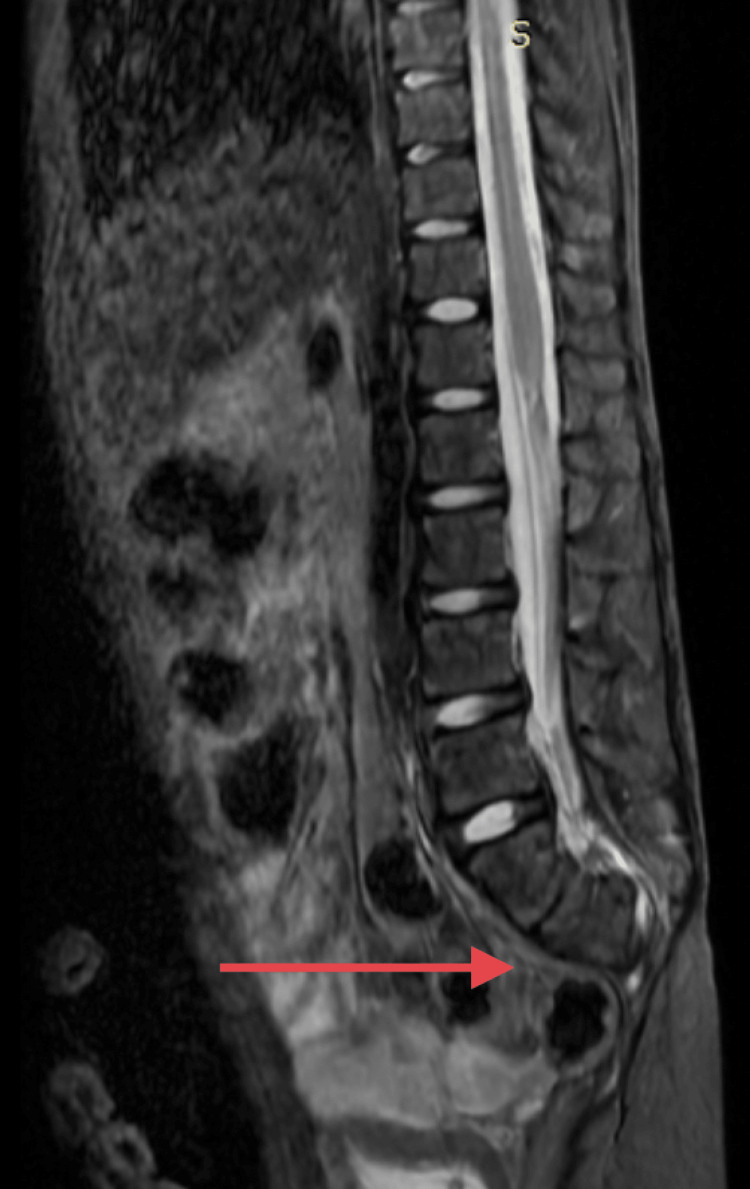
MRI spine sagittal section showing absent sacral vertebrae below the level of the second sacral vertebra MRI: magnetic resonance imaging

Blood investigations revealed a hemoglobin level of 7.7 g/dL. Other aspects of complete blood count including total counts and platelets were within normal limits. Renal function tests showed a urea level of 64 mg/dl and her creatinine level was 1.7 mg/dl, indicating renal injury. A radiograph was performed, which showed an absent ala on the left side of the sacrum and no sacrum below the level of the second sacral vertebra. An abdominal ultrasonography was then performed, which showed the presence of medical renal disease most likely due to urinary reflux. The patient was then scheduled to undergo an MRI of the lower spine and pelvis for further assessment. The MRI findings confirmed our suspicion of SA. The MRI showed absent sacral ala on the left side with absent sacral vertebrae below the level of the second sacral vertebra (Figure 1). The ilia was articulating with the first sacral vertebra. There was also a straightening of the lumbar spine and spina bifida at the level of L5-S1. These findings were consistent with Renshaw type II SA. There was also a terminal filar lipoma at the level of the first lumbar vertebra. The MRI also showed a contracted left kidney. Micturating cystourethrogram showed the presence of bilateral reflux.

Management

The patient was treated with the aid of the pediatric surgery team. Clean intermittent self-catheterization (CIC) was taught to the patient and her parents. The patient's urea and creatinine values decreased but did not normalize. Constipation was treated with laxatives and dietary advice was provided. The patient was counseled about the presence of renal disease and risk of progression to renal failure and the subsequent need for dialysis. She was advised regular follow-ups every month with ultrasonography to monitor the progression of renal disease and to assess the need for surgical management of urinary incontinence. One of the key takeaways from the case was the psychological impact the disease had caused the patient and her parents. A pediatric psychologist was involved during the treatment process. Identifying the cause of her symptoms led to perceptible relief for the patient and her family. She was diagnosed with anxiety and depression. Interpersonal therapy (IPT) was subsequently initiated and the family was supportive during therapy.

## Discussion

SA is a rare condition characterized by the absence of one or more lower sacral vertebral bodies. The term "sacral agenesis" encompasses a spectrum of anomalies and is considered a part of the CRS spectrum. The clinical presentation can vary from subtle skeletal abnormalities to severe neurological deficits, impacting the patient's quality of life. SA is a well-known component of many syndromes. One of the most common associations is the Currarino triad.

The dominantly inherited Currarino triad or syndrome is characterized by the triad of SA abnormalities (abnormally developed lower spine), anorectal malformation (most commonly in the form of anorectal stenosis), and presacral mass consisting of a teratoma, anterior sacral meningocele or both. Currarino triad has been associated with mutations in the MNX1 gene (previously HLXB9). However, MNX1 mutations account for only 50% of sporadic and 90% of familial cases [3]. Newer studies have revealed that diabetes-relevant (CLTCL1 and PDZD2) or pancreatic-expressed (MORN1 and ZNF330) genes are also associated with SA and CRS. Most genetic studies have been low on sample size, but it is assumed that the presence of diabetes-associated genes is a likely cause of the association of SA with maternal diabetes [4]. Other associated syndromes include VACTERL (comprising vertebral anomalies, anal atresia, cardiac anomalies, tracheoesophageal fistula, renal anomalies, and limb anomalies), OEIS (characterized by omphalocele, cloacal exstrophy, imperforate anus, and spinal defects), and the Currarino triad [5]. 

Our patient's mother had a history of maternal diabetes that was poorly controlled. The history was obtained only orally and no documents were not available to substantiate it. There was no prenatal exposure to other teratogens. The etiology of SA is under a lot of debate. It is now assumed that early embryonic insult before four weeks of gestation to the mid-posterior axial mesoderm is responsible for SA and CRS. CRS represents a spectrum of congenital malformations of the spine. It can range from agenesis of the lumbosacral spine to the most severe cases of sirenomelia associated with lower extremity fusion and major vessel anomalies [2].

Renshaw classified SA into four types, as shown in Table 1. The Renshaw classification is based on the vertebral agenesis severity and articulation of remaining vertebrae to the iliac bone [6].

**Table 1:** Renshaw classification of sacral agenesis* *[6]

Renshaw Type	Partial/Complete	Description
Type I	Partial	Partial or total unilateral sacral agenesis
Type II	Partial	Partial sacral agenesis with bilaterally symmetrical defect, a normal or hypoplastic sacral vertebra, and a stable articulation between the ilia and first sacral vertebra
Type III	Complete	Variable lumbar and total sacral agenesis, with the ilia articulating with the sides of the lowest vertebra present
Type IV	Complete	Variable lumbar and total sacral agenesis, with the caudal endplate of the lowest vertebra resting above either fused ilia or an iliac amphiarthrosis

Vertebral column termination can occur at any level, from T8−9 to only involving the coccyx. Usually, one or two vertebrae distal to the lowest intact vertebrae are hypoplastic [7]. There is usually no correlation between the level of bony abnormalities and neurological deficits. The motor deficit is usually more pronounced than the sensory deficit. This implies that in spite of intact perianal sensation, there may be bladder or anal sphincter involvement. This notable incongruity between motor and sensory deficits is typical. While the exact causes are unclear, it is postulated that sensory functions are spared because of the detachment of the dorsal ganglia from the neural tube in early development [5].

As in our case, patients often present with dribbling of urine and chronic urinary infection secondary to vesicoureteric reflux (VUR) without any obvious physical signs that suggest SA. Detailed clinical examination can lead to observations such as flattened gluteal cleft, gluteal dimpling, a sacral lipoma, myelomeningocele, or orthopedic anomalies, which could point to a diagnosis of SA and CRS. An anteroposterior radiograph of the spine is usually sufficient to diagnose SA.

Antenatal ultrasound is key to early diagnosis of SA and CRS. Fetal crown-rump length in the first trimester is reported to be shorter than in a normal fetus but is not very diagnostic [2]. Calcification of the sacrum is usually completed by 16 weeks of gestation. This particular observation allows us to accurately exclude isolated SA at 16-17 weeks of gestation when the S1-S2 ossification nuclei are visualized. This is of significance in diabetic mothers in whom the risk is high [8]. In rural India, lack of expertise in antenatal screening is a major challenge that leads to a huge number of missed diagnoses. 

Bowel and bladder disturbances are common features in SA. Patients who present late often come to the pediatric OPD with bladder disturbances and signs of neurogenic bladder. SA is associated with neurogenic bladder dysfunction in 80% of cases. A comprehensive study at the Boston Children’s Hospital using urodynamic studies showed that 52% of children with SA had upper motor neuron (UMN) lesions and 16% had lower motor neuron (LMN) lesions. Of note, 13% had a mixed lesion type. This study involved 20 children. An interesting observation was that 30% (six children) revealed a significant change in neurologic status: no deficit to UMN (two), UMN to no deficit (two), and mixed to LMN (two) [9].

Urodynamic studies are a key component of the assessment of these patients. It also helps to determine if a patient’s current bladder management is appropriate. With early diagnosis the norm in most parts of the world, end-stage renal disease (ESRD) is rare. However, in areas with poor access to medical resources, ESRD is not uncommon. Many of these children progress to ESRD, requiring dialysis at some point in their lives. In our setting, the lack of availability of pediatric urodynamic testing is a major impediment. Most cases do well with CIC. When the urinary tract is closely monitored, children are less likely to develop hydronephrosis or VUR. Constant and careful monitoring is therefore essential to assess the need for vesicostomy or ureteric reimplantation. The need for ureteric reimplantation or vesicostomy is also higher in patients who present late [9]. Constipation is a common feature in SA and CRS. Interestingly, 3% of children with intractable constipation have been found to have lumbosacral spine abnormalities, even in the absence of neurologic symptoms [10]. In our case, the delayed diagnosis meant that a psychosomatic cause was attributed. Anxiety and depression were noted in the patient. In the modern era, depression and anxiety in children are recognised as major concerns and numerous methodologies have been adopted to treat the same. IPT is an accepted treatment modality for childhood anxiety and depression. This therapeutic approach follows a structured manual, operates within a specific time frame, and places emphasis on addressing the reciprocal relationship between a child's emotional state and their present relationships as the central elements of treatment [11].

## Conclusions

This case report about a patient who presented with chronic constipation and neurogenic bladder and was diagnosed with renal disease highlights the need for early imaging in children who present with such symptoms. The role of expert antenatal screening in diabetic mothers is also important. The psychological impact of delayed diagnosis of congenital conditions can be debilitating to the child and family. A thorough understanding of SA and CRS among pediatricians and healthcare workers could possibly prevent long-term complications. A multidisciplinary team involving pediatricians, pediatric surgeons, and child psychologists is crucial to provide comprehensive care for these patients.
